# Cigarette smoking, genetic polymorphisms and colorectal cancer risk: the Fukuoka Colorectal Cancer Study

**DOI:** 10.1186/1471-2407-10-274

**Published:** 2010-06-10

**Authors:** Hoirun Nisa, Suminori Kono, Guang Yin, Kengo Toyomura, Jun Nagano, Ryuichi Mibu, Masao Tanaka, Yoshihiro Kakeji, Yoshihiko Maehara, Takeshi Okamura, Koji Ikejiri, Kitaroh Futami, Takafumi Maekawa, Yohichi Yasunami, Kenji Takenaka, Hitoshi Ichimiya, Reiji Terasaka

**Affiliations:** 1 Department of Preventive Medicine, Graduate School of Medical Sciences, Kyushu University, 3-1-1 Maidashi, Higashi-ku, Fukuoka, 812-8582, Japan; 2 Department of Surgery and Oncology, Graduate School of Medical Sciences, Kyushu University, 3-1-1 Maidashi, Higashi-ku, Fukuoka, 812-8582, Japan; 3 Department of Surgery and Science, Graduate School of Medical Sciences, Kyushu University, 3-1-1 Maidashi, Higashi-ku, Fukuoka, 812-8582, Japan; 4 Department of Gastroenterological Surgery, National Kyushu Cancer Center, 3-1-1 Notame, Minami-ku, Fukuoka, 811-1395, Japan; 5 Division of Surgery, National Kyushu Medical Center, 1-8-1 Jigyohama, Chuo-ku, Fukuoka, 810-8563, Japan; 6 Department of Surgery, Fukuoka University Chikushi Hospital, 377-1 Oaza-zokumyoin, Chikushino-shi, 818-0067, Japan; 7 Department of Regenerative Medicine and Transplantation, Faculty of Medicine, Fukuoka University, 7-45-1 Nanakuma, Jonan-ku, Fukuoka, 814-0180, Japan; 8 Division of Surgery, Fukuoka City Hospital, School of Medicine, 13-1 Yoshizuka-honmachi, Hakata-ku, Fukuoka, 812-0046, Japan; 9 Division of Surgery, Hamanomachi General Hospital, 3-5-27 Maizuru, Chuo-ku, Fukuoka, 810-8539, Japan; 10 Division of Surgery, Fukuoka Red Cross Hospital, 3-1-1 Ogusu, Minami-ku, Fukuoka, 815-8555, Japan

## Abstract

**Background:**

It is uncertain whether smoking is related to colorectal cancer risk. Cytochrome P-450 CYP1A1, glutathione-S-transferase (GST) and NAD(P)H:quinone oxidoreductase 1 (NQO1) are important enzymes in the metabolism of tobacco carcinogens, and functional genetic polymorphisms are known for these enzymes. We investigated the relation of cigarette smoking and related genetic polymorphisms to colorectal cancer risk, with special reference to the interaction between smoking and genetic polymorphism.

**Methods:**

We used data from the Fukuoka Colorectal Cancer Study, a population-based case-control study, including 685 cases and 778 controls who gave informed consent to genetic analysis. Interview was conducted to assess lifestyle factors, and DNA was extracted from buffy coat.

**Results:**

In comparison with lifelong nonsmokers, the odds ratios (OR) of colorectal cancer for <400, 400-799 and ≥800 cigarette-years were 0.65 (95% confidence interval [CI], 0.45-0.89), 1.16 (0.83-1.62) and 1.14 (0.73-1.77), respectively. A decreased risk associated with light smoking was observed only for colon cancer, and rectal cancer showed an increased risk among those with ≥400 cigarette-years (OR 1.60, 95% CI 1.04-2.45). None of the polymorphisms under study was singly associated with colorectal cancer risk. Of the gene-gene interactions studied, the composite genotype of CYP1A1*2A or CYP1A1*2C and *GSTT1 *polymorphisms was associated with a decreased risk of colorectal cancer, showing a nearly statistically significant (*P*_interaction _= 0.06) or significant interaction (*P*_interaction _= 0.02). The composite genotypes of these two polymorphisms, however, showed no measurable interaction with cigarette smoking in relation to colorectal cancer risk.

**Conclusions:**

Cigarette smoking may be associated with increased risk of rectal cancer, but not of colon cancer. The observed interactions between *CYP1A1 *and *GSTT1 *polymorphisms warrant further confirmation.

## Background

Both environmental and genetic factors are thought to play an important role in colorectal carcinogenesis [1]. The role of genetic factors in the etiology of colorectal cancer is estimated to be 35% in a twin study [2]. Recent genome-wide association studies have identified several novel single nucleotide polymorphisms (SNPs) associated with colorectal cancer risk, suggesting the importance of combination of low-penetrance genes [3]. Furthermore, it is estimated that one SNP is involved in approximately 15% of colorectal cancer in European populations [4].

A large number of studies have consistently shown that cigarette smoking is associated with increased risk of colorectal adenoma, a well-established precursor lesion of colorectal cancer, as reviewed elsewhere [5,6]. The findings on smoking and colorectal cancer are inconsistent, however. While a recent meta-analysis reported a statistically significant 1.18-fold increase in the risk of colorectal cancer associated with smoking [7], individual studies showed a weak or null association between smoking and colorectal cancer [5,7]. For example, several studies suggested a modest increase in the risk of colorectal cancer associated with smoking [8-11], but other studies failed to find such a positive association [12-14].

Tobacco smoke contains various types of carcinogens such as polycyclic aromatic hydrocarbons (PAHs), heterocyclic amines, aromatic amines and N-nitrosamines which require metabolic activation and detoxification by different enzymatic pathways, including cytochrome P-450 (CYP), glutathione-S- transferases (GSTs) and NAD(P)H:quinone oxidoreductase 1 (NQO1). CYP1A1 is a phase I, predominantly extrahepatic, microsomal enzyme involved in the bioactivation of PAHs including benzo(a)pyrene. Two functional polymorphisms are known in the CYP1A1 gene; one is 3698T>C substitution (CYP1A1*2A, rs 4646903) creating an *Msp*I restriction site in the 3'-flanking region, and the other is 2454A>G substitution (CYP1A1*2C, rs 1048943) resulting in an amino acid change in exon 7 (Ile462Val) [15]. The CYP1A1*2A and CYP1A1*2C alleles are putatively linked to higher inducibility of the enzyme, and some studies have suggested an increased risk of tobacco-related cancers associated with these variant alleles [15,16]. An increased risk of *in situ *colorectal carcinoma associated with CYP1A1*2A was reported in a small case-control study in Hawaii [17], but no association between CYP1A1*2A and colorectal cancer was observed in subsequent studies [18-20]. CYP1A1*2C was unrelated to colorectal cancer risk in these studies [17-20], but was associated with an increased risk in another study [21]. GSTs are a superfamily of detoxification enzymes that facilitate the inactivation of chemical carcinogens and environmental toxic compounds [22]. GSTs consist of several classes of genes, and *GSTM1 *and *GSTT1 *polymorphisms have been investigated most intensively in relation to tobacco-related cancers [22]. The null genotypes of these polymorphisms result in a complete loss of enzyme function and thus may be at increased risk of tobacco-related cancers [22]. Results on *GSTM1 *and *GSTT1 *polymorphisms in relation to colorectal cancer are inconsistent as reviewed elsewhere [23,24]. The *GSTP1 *gene also has a functional polymorphism, but this polymorphism is unlikely to play an important role for smoking-related cancers [25]. The NQO1 is involved in detoxification through their two electron reduction to hydroquinones, thereby inhibiting the DNA adduct formation although NQO1 can act as pro-oxidant in certain conditions [26]. The functional 609C>T polymorphism (rs 1800566) causing amino acid change (Pro187Ser) results in loss of NQO1 activity, and may increase susceptibility to the risk of cancer, especially of tobacco-related cancers [26]. A meta-analysis suggested an increased susceptibility to colorectal cancer as well as lung and bladder cancers associated with *NQO1 *187Ser allele, although the results from individual studies were heterogeneous [27].

Previously, several studies have addressed the interaction between cigarette smoking and one or more of these polymorphisms on colorectal cancer risk [19,20,28-33], and some suggested an interaction between *GSTT1 *or *GSTM1 *null genotype and cigarette smoking [28,30] and between CYP1A1*2A or CYP1A1*2C and cigarette smoking [19]. Few studies have addressed the gene-gene interaction between phase I and II enzymes in relation to colorectal carcinogenesis [20,21,34]. In the present study, we examined the relation of *CYP1A1*, *GSTM1*, *GSTT1 *and *NQO1 *polymorphisms as well as of cigarette smoking to colorectal cancer risk in the Fukuoka Colorectal Cancer Study, a community-based case-control study [35], focusing on the interaction with cigarette smoking and gene-gene interaction. This is the first study regarding combined genotypes of phase I and II enzymes and colorectal cancer risk in Japan.

## Methods

The Fukuoka Colorectal Cancer Study is a case-control study of incident cases and community controls in Fukuoka City and three adjacent areas. Details of methodological issues have been described elsewhere [35]. The study protocol was approved by the ethics committees of the Kyushu University, Faculty of Medical Sciences and of all except two of the participating hospitals. The two hospitals had no ethics committees at the time of survey, and approval was obtained from the director of each hospital.

### Subjects

Cases were a consecutive series of patients with histologically confirmed incident colorectal adenocarcinoma who were admitted to one of the participating hospitals (two university hospitals and six affiliated hospitals) for surgical treatment during the period September 2000 to December 2003. Eligible cases were Japanese men and women aged 20 to 74 years at time of diagnosis; lived in the study area; had no prior history of partial or total removal of the colorectum, familial adenomatous polyposis, or inflammatory bowel disease; and were mentally competent to give informed consent and to complete the interview. Of the total 1,053 eligible cases, 840 (80%) cases participated in the interview, and 685 gave informed consent for the genotyping.

Controls were randomly selected from the study area by frequency-matching with respect to gender and 10-year age group. Eligibility criteria for controls were the same as described for the cases except that they had no prior diagnosis of colorectal cancer. A total of 1,500 persons were selected as control candidates by a two-stage random sampling, using residential registry. They were invited to participate in the study by mail. Of these, 833 persons participated in the survey, and 778 gave an informed consent for the genotyping. The participation rate for the interview was calculated as 60% (833 of 1,382), after exclusion of 118 persons for the following reasons: death (n = 7), migration from the study area (n = 22), undelivered mail (n = 44), mental incompetence (n = 19), history of partial or total removal of the colorectum (n = 21) and diagnosis of colorectal cancer after the survey (n = 5).

### Interview

Research nurses interviewed cases and controls in person regarding smoking, alcohol intake, physical activity and other factors using a uniform questionnaire. Interviews for cases were conducted in hospital during admission, and those for controls were conducted mostly at public community centers or collaborating clinics. The referent time for cases was the date of the onset of symptoms or the screening, and that for controls was the time of interview. Detailed information on smoking history was ascertained by asking individuals firstly whether they had ever smoked cigarettes daily for one year or longer. Age of starting smoking and that of quitting smoking (for past smokers) were ascertained, along with years of smoking and numbers of cigarettes smoked per day for each decade of age from the second to eighth decade. Cumulative exposure to cigarette smoking until the beginning of the previous decade of age was expressed by cigarette-years, the number of cigarettes smoked per day multiplied by years of smoking, and classified into 0, 1-399, 400-799 and >800 cigarette-years.

Alcohol consumption at the time of five years prior to the referent time was elicited. The amount of alcohol was expressed in the conventional unit; one *go *(180 mL) of *sake*, one large bottle (633 mL) of beer and half a *go *(90 mL) of *shochu *were each expressed as one unit; and one drink (30 mL) of whisky or brandy and one glass (100 mL) of wine were each converted to a half unit. Questions on physical activities elicited type of job (sedentary or standing work, work with walking, labor walk, hard labor work and no job), activities in commuting and housework, together with leisure-time activities at the time five years previously. As described in detail previously [36], leisure-time physical activity (including activities in commuting and housework) was expressed as a sum of metabolic equivalents (MET) multiplied by hours of weekly participation in each activity.

Height (cm), recent body weight and body weight at the time 10 years before were elicited. Body mass index (kg/m^2^) 10 years earlier was used because the current body mass index was unrelated to risk [36]. Body weight 10 years earlier was not ascertained from 2 cases and 10 controls and was substituted with the current body weight.

### Genotyping

DNA was extracted from the buffy coat by using a commercial kit (Qiagen GmbH, Hilden, Germany), and genotyping was performed using the PCR-RFLP or PCR method. The PCR was performed in a reaction mixture of 10 μL containing approximately 50-150 ng/μL. Genotyping for the CYP1A1*2A polymorphism was analyzed by PCR-RFLP using primers 5'-TAGGA GTCTT GTCTC ATGCC T-3' (sense) and 5'-CAGTG AAGAG GTGTA GCCGC T-3' (anti-sense) [17]. The PCR product of 340 bp was digested with *Msp*I, resulting in fragments of 200 and 140 bp for the CYP1A1*2A allele. The CYP1A1*2C polymorphism was determined by the PCR-RFLP method using primers 5'-GAACT GCCAC TTCAG CTGTC T-3' (sense) and 5'-GAAAG ACCTC CCAGC GGTCA-3' (anti-sense) [37]. The PCR product of 187 bp was cleaved into three fragments (120, 48 and 19 bp) in the presence of the CYP1A1*2C allele, and otherwise into two fragments (139 and 48 bp). *GSTM1 *and *GSTT1 *polymorphisms were determined by the multiplex PCR method using primers for *GSTM1*, *GSTT1 *and albumin as described elsewhere [38]. Genotyping for *NQO1 *Pro187Ser was performed as described earlier [39], using primers 5'-TCTCC TCATC CTGTA CCTCT-3' (sense) and 5'-TCCTC AGAGT GGCAT TCTGC-3' (anti-sense). The PCR product of 230 bp was digested with *Hinf*I, resulting in fragments of 195 and 35 bp for the 187Pro allele, and in fragments of 151, 44 and 35 bp for the 187Ser allele.

### Statistical analysis

Associations of cigarette smoking and genetic polymorphisms with colorectal cancer were examined in terms of odds ratio (OR) and 95% confidence interval (CI) which were obtained from a logistic regression analysis. The multivariate models always included indicator variables for 5-year age class (starting with the lowest class of <50 years), sex, residence area (Fukuoka City or the adjacent areas), body mass index 10 years ago (<22.5, 22.5-24.9, 25.0-27.4 or ≥27.5 kg/m^2^), smoking (0, 1-399, 400-799 or ≥800 cigarettes-years), alcohol intake (0, 0.1-0.9, 1.0-1.9 or ≥2.0 units/day), type of job (sedentary, moderate or hard), leisure-time physical activity (0, 1-15.9 or ≥16 MET-hours/week) and parental history of colorectal cancer.

Gene-gene and gene-environment (smoking) interactions were statistically evaluated based on the likelihood test, comparing the model including a term or terms for interaction and the model without. Deviation from the Hardy-Weinberg equilibrium was evaluated by chi-square test with 1 degree of freedom. Statistical significance was declared if a two-sided *P*-value was less than 0.05. Statistical analyses were carried out using SAS version 9.2 (SAS Institute, Cary, NC).

## Results

Table 1 shows the association between cigarette smoking and colorectal cancer risk. Adjustment for the covariates did not markedly change the results. As compared with lifelong nonsmokers, men and women with a light exposure to cigarette smoking (1-399 cigarette-years) showed a moderate decrease in the OR of colorectal cancer. The decreases in the OR in both sexes combined and in women were statistically significant. The ORs for higher categories of smoking were slightly greater than unity in both men and women, but the increases were not statistically significant. In men and women combined, the multivariate-adjusted ORs for past and current smokers as compared with lifelong nonsmokers were 0.90 (95% CI 0.66-1.24) and 0.80 (95% CI 0.58-1.05), respectively. There was no clear association between cumulative years of smoking and colorectal cancer; the multivariate-adjusted ORs for 0, 1-14, 15-29 and ≥30 years of smoking were 1.00 (referent), 0.73 (0.51-1.05), 0.92 (0.65-1.29) and 1.02 (0.69-1.49), respectively. Cases of colon and rectal cancer numbered 384 and 290, respectively; 11 cases had both colon and rectal cancers. The multivariate-adjusted ORs of colon cancer for 0, 1-399, 400-799 and 800 cigarette-years were 1.00 (referent), 0.49 (95% CI 0.32-0.73), 0.89 (0.60-1.33) and 1.02 (0.62-1.69), respectively, while the corresponding values for rectal cancer were 1.00 (referent), 0.92 (0.60-1.42), 1.72 (1.10-2.66) and 1.23 (0.66-2.26), respectively. The OR of rectal cancer for the highest two categories combined was 1.60 (95% CI 1.04-2.45).

**Table 1:** Risk of colorectal cancer according to cigarette smoking

	Number (%)			
			
Cigarette-years	Cases	Controls	OR (95% CI)*	OR (95% CI)^†^
Both sexes				
0	299 (43.6)	326 (41.9)	1.00 (referent)	1.00 (referent)
1-399	117 (17.1)	201 (25.8)	0.68 (0.50-0.94)	0.65 (0.47-0.89)
400-799	195 (28.5)	180 (23.1)	1.21 (0.88-1.68)	1.16 (0.83-1.62)
≥800	74 (10.8)	71 (9.1)	1.21 (0.79-1.86)	1.14 (0.73-1.77)
Men				
0	80 (18.8)	92 (18.8)	1.00 (referent)	1.00 (referent)
1-399	94 (22.1)	158 (32.2)	0.73 (0.49-1.09)	0.69 (0.46-1.04)
400-799	182 (42.7)	171 (34.9)	1.24 (0.85-1.81)	1.15 (0.78-1.71)
≥800	70 (16.4)	69 (14.1)	1.16 (0.72-1.87)	1.05 (0.64-1.71)
Women				
0	219 (84.6)	234 (81.3)	1.00 (referent)	1.00 (referent)
1-399	23 (8.9)	43 (14.9)	0.55 (0.32-0.95)	0.48 (0.27-0.86)
≥400	17 (6.6)	11 (3.8)	1.59 (0.72-3.51)	1.54 (0.67-3.55)

* Adjusted for sex, age, and residence area.

† Adjusted for sex, age, residence area, alcohol consumption, body mass index, type of job, leisure-time physical activity and parental colorectal cancer.

None of the five polymorphisms showed a measurable association with the risk of colorectal cancer, nor did the composite genotype of *GSTM1 *and *GSTT1 *(Table 2). Each polymorphism was not associated with smoking history (cigarette-years) in either men or women among controls (data not shown). Frequencies of CYP1A1*2A allele were 0.363 in cases and 0.372 in controls, and frequencies of CYP1A1*2C allele were 0.221 in cases and 0.230 in controls. Frequencies of the 187Ser allele of *NQO1 *polymorphism were 0.376 in cases and 0.385 in controls. Genotype distributions of these three polymorphisms were in accordance with the Hardy-Weinberg equilibrium within each cases and controls (all *P *>0.05). CYP1A1*2A and CYP1A1*2C polymorphisms were in complete linkage disequilibrium.

**Table 2:** Risk of colorectal cancer in relation to selected genetic polymorphisms

	Number (%)		
		
Genotype	Cases, n = 685	Controls, n = 778	OR (95% CI)*
CYP1A1*2A			
0†	283 (41.3)	305 (39.2)	1.00 (referent)
1	307 (44.8)	368 (47.3)	0.90 (0.72-1.13)
2	95 (13.9)	105 (13.5)	0.97 (0.70-1.35)
CYP1A1*2C			
0†	418 (61.0)	461 (59.3)	1.00 (referent)
1	231 (33.7)	276 (35.5)	0.94 (0.75-1.17)
2	36 (5.3)	41 (5.3)	1.00 (0.62-1.62)
*GSTM1*			
Non-null	328 (47.9)	356 (45.8)	1.00 (referent)
Null	357 (52.1)	422 (54.2)	0.90 (0.73-1.11)
*GSTT1*			
Non-null	347 (50.7)	435 (55.9)	1.00 (referent)
Null	338 (49.3)	343 (44.1)	1.20 (0.97-1.48)
*GSTM1 *+ *GSTT1*			
Non-null	502 (73.3)	589 (75.7)	1.00 (referent)
Null	183 (26.7)	189 (24.3)	1.09 (0.86-1.39)
*NQO1 *Pro187Ser‡			
Pro/Pro	259 (37.9)	282 (36.3)	1.00 (referent)
Pro/Ser	336 (49.1)	392 (50.5)	0.94 (0.75-1.18)
Ser/Ser	89 (13.0)	103 (13.3)	0.97 (0.69-1.35)

* Adjusted for sex, age, residence area, cigarette smoking, alcohol consumption, body mass index, type of job, leisure-time physical activity and parental colorectal cancer.

† Number of the variant allele.

‡ One case and one control were excluded because of undetermined genotype.

There was no material interaction between cigarette smoking and each polymorphism on colorectal cancer risk (Table 3). Repeated analyses for men and for colon and rectal cancers did not show any measurable interaction between smoking and genotype.

**Table 3:** Effect modification of cigarette smoking on colorectal cancer risk associated with selected genetic polymorphisms

	< 400		≥ 400		
		
Genotype	n*	OR (95% CI)†	n*	OR (95% CI)†	*P *for interaction
CYP1A1*2A					
0‡	161/207	1.00 (referent)	122/98	1.76 (1.19-2.59)	0.14
≥1	255/320	1.04 (0.79-1.36)	147/153	1.30 (0.91-1.87)	
CYP1A1*2C					
0‡	243/309	1.00 (referent)	175/152	1.58 (1.14-2.20)	0.30
≥1	173/218	1.03 (0.79-1.34)	94/99	1.28 (0.87-1.86)	
*GSTM1*					
Non-null	207/245	1.00 (referent)	121/111	1.38 (0.96-1.97)	0.68
Null	209/282	0.88 (0.67-1.14)	148/140	1.33 (0.94-1.87)	
*GSTT1*					
Non-null	211/301	1.00 (referent)	136/134	1.51 (1.08-2.13)	0.60
Null	205/226	1.24 (0.95-1.61)	133/117	1.67 (1.18-2.37)	
*GSTM1*+*GSTT1*					
Non-null	304/407	1.00 (referent)	198/182	1.53 (1.14-2.07)	0.29
Null	112/120	1.20 (0.89-1.63)	71/69	1.41 (0.94-2.11)	
*NQO1 *Pro187Ser§					
Pro/Pro	157/189	1.00 (referent)	102/93	1.41 (0.95-2.09)	0.89
Pro/Ser + Ser/Ser	259/338	0.94 (0.72-1.24)	166/157	1.37 (0.96-1.94)	

* Numbers of cases/controls.

† Adjusted for sex, age, residence area, alcohol consumption, body mass index, type of job, leisure-time physical activity and parental colorectal cancer.

‡ Number of the variant allele.

§ One case and one control were excluded because of undetermined genotype.

We further examined gene-gene interactions for the combination of *CYP1A1 *and *GST *polymorphisms (Table 4) and *CYP1A1 *and *NQO1 *polymorphisms (Table 5). The combination of CYP1A1*2A or CYP1A1*2C and *GSTT1 *polymorphisms showed a nearly statistically significant or significant interaction. The composite genotype of *GSTT1 *non-null and CYP1A1*2A or CYP1A1*2C allele was associated with a decreased risk of colorectal cancer (Table 4). There was no measurable interaction between *CYP1A1 *and *NQO1 *polymorphisms in relation to colorectal cancer risk (Table 5). Decreased risks for the combination of *CYP1A1 *variant allele and *GSTT1 *non-null genotype were observed only in men; the multivariate-adjusted ORs were 0.62 (95% CI 0.42-0.90) for the combination of CYP1A1*2A allele and *GSTT1 *non-null genotype (*P*_interaction _= 0.04) and 0.63 (95% CI 0.43-0.91) for that of CYP1A1*2C allele and *GSTT1 *non-null genotype (*P*_interaction _= 0.07).

**Table 4:** Combinations of *CYP1A1 *and *GST *polymorphisms and colorectal cancer risk

		Number (%)			
				
Combination of genotypes		Cases	Controls	OR (95% CI)*	*P *for interaction
CYP1A1*2A	*GSTM1*				
0†	Non-null	122 (17.8)	135 (17.4)	1.00 (referent)	
≥1	Non-null	206 (30.1)	221 (28.4)	1.02 (0.74-1.40)	0.34
0	Null	161 (23.5)	170 (21.9)	1.01 (0.72-1.42)	
≥1	Null	196 (28.6)	252 (32.4)	0.84 (0.61-1.15)	
CYP1A1*2A	*GSTT1*				
0†	Non-null	147 (21.5)	158 (20.3)	1.00 (referent)	
≥1	Non-null	200 (29.2)	277 (35.6)	0.75 (0.56-1.01)	0.06
0	Null	136 (19.9)	147 (18.9)	0.93 (0.67-1.30)	
≥1	Null	202 (29.5)	196 (25.2)	1.07 (0.79-1.45)	
CYP1A1*2A	*GSTT1 *+ *GSTM1*				
0†	Non-null	203 (29.6)	222 (28.5)	1.00 (referent)	
≥1	Non-null	299 (43.6)	367 (47.2)	0.88 (0.69-1.13)	0.51
0	Null	80 (11.7)	83 (10.7)	0.99 (0.68-1.43)	
≥1	Null	103 (15.0)	106 (13.6)	1.03 (0.73-1.44)	
CYP1A1*2C	*GSTM1*				
0†	Non-null	187 (27.3)	207 (26.6)	1.00 (referent)	
≥1	Non-null	141 (20.6)	149 (19.2)	1.06 (0.78-1.45)	0.29
0	Null	231 (33.7)	254 (32.6)	0.98 (0.75-1.29)	
≥1	Null	126 (18.4)	168 (21.6)	0.83 (0.60-1.13)	
CYP1A1*2C	*GSTT1*				
0†	Non-null	214 (31.2)	239 (30.7)	1.00 (referent)	
≥1	Non-null	133 (19.4)	196 (25.2)	0.76 (0.56-1.01)	0.02
0	Null	204 (29.8)	222 (28.5)	0.98 (0.75-1.29)	
≥1	Null	134 (19.6)	121 (15.6)	1.23 (0.90-1.69)	
CYP1A1*2C	*GSTT1 *+ *GSTM1*				
0†	Non-null	300 (43.8)	337 (43.3)	1.00 (referent)	
≥1	Non-null	202 (29.5)	252 (32.4)	0.90 (0.70-1.15)	0.39
0	Null	118 (17.2)	124 (15.9)	1.00 (0.74-1.36)	
≥1	Null	65 (9.5)	65 (8.4)	1.13 (0.77-1.66)	

* Adjusted for sex, age, residence area, cigarette smoking, alcohol consumption, body mass index, type of job, leisure-time physical activity and parental colorectal cancer.

† Number of the variant allele.

**Table 5:** Combinations of *CYP1A1 *and *NQO1 *polymorphisms and colorectal cancer risk

		**Number (%)**			
				
**Combination of genotypes**		**Cases**	**Controls**	OR (95% CI)*	***P *for interaction**
CYP1A1*2A	*NQO1 *Pro187Ser†				
0‡	Pro/Pro	126 (18.4)	118 (15.2)	1.00 (referent)	
≥1	Pro/Pro	133 (19.4)	164 (21.1)	0.78 (0.55-1.10)	0.22
0	Pro/Ser + Ser/Ser	157 (23.0)	187 (24.1)	0.81 (0.58-1.14)	
≥1	Pro/Ser + Ser/Ser	268 (39.2)	308 (39.6)	0.83 (0.61-1.13)	
CYP1A1*2C	*NQO1 *Pro187Ser†				
0‡	Pro/Pro	162 (23.7)	176 (22.7)	1.00 (referent)	
≥1	Pro/Pro	97 (14.2)	106 (23.6)	1.04 (0.73-1.48)	0.53
0	Pro/Ser + Ser/Ser	256 (37.4)	285 (36.7)	1.00 (0.76-1.32)	
≥1	Pro/Ser + Ser/Ser	169 (24.7)	210 (27.0)	0.90 (0.67-1.22)	

* Adjusted for sex, age, residence area, cigarette smoking, alcohol consumption, body mass index, type of job, leisure-time physical activity and parental colorectal cancer.

† One case and one control were excluded because of undetermined genotype.

‡ Number of the variant allele.

Cigarette smoking showed no effect modification on associations with composite genotypes of *CYP1A1 *and *GST *or *NQO1 *polymorphisms. For example, the decreased risk among individuals harboring CYP1A1*2A or CYP1A1*2C allele and *GSTT1 *non-null genotype was observed regardless of exposure to smoking. In other words, high exposure to smoking was consistently related to an increased risk of colorectal cancer across different composite genotypes (see additional file 1).

## Discussion

Many studies have addressed the association between cigarette smoking and colorectal cancer risk, and their findings are highly variable although an 18% increase in colorectal cancer risk was estimated for ever-smokers versus never-smokers in a recent meta-analysis [7]. The variable results may be due to differences in study method, statistical power and ethnicity. The association may differ by sex or location of colorectal cancer. In fact, prospective studies showed higher risk estimates than case-control studies in the meta-analysis [7]. Furthermore, while an increased risk associated with smoking was observed in both men and women, the positive association with smoking was more evident for rectal cancer than for colon cancer [7]. The present finding adds to evidence that cigarette smoking is associated with increased risk of rectal cancer. It was unexpected that individuals with an exposure of 1-399 cigarette-years had a decreased risk of colorectal cancer. This decrease was observed for colon cancer but not for rectal cancer, and was more marked in women. Previously, some case-control studies also suggested that smoking was associated with a decreased risk of distal colon cancer in Caucasians [40] and of colon cancer in Japanese [41,42]. We have no clear explanation to the decreased risk of colon cancer associated with light smoking although confounding remains a possible explanation.

In agreement with the results from three studies [18-20], the present study did not show an association of either CYP1A1*2A or CYP1A1*2C polymorphism with colorectal cancer risk. An 8-fold increased risk of colorectal cancer among Japanese homozygotes of CYP1A1*2A allele in Hawaii is probably a chance finding due to small numbers (23 cases and 59 controls) [17]. Of these previous studies, two examined the interaction between *CYP1A1 *polymorphisms and smoking [19,20]. One study reported an increased risk of rectal cancer, but not of colon cancer, among former and current smokers who did not carry either CYP1A1*2A or CYP1A1*2C allele [19], while the other study showed no interaction between either of the *CYP1A1 *polymorphisms and smoking on colorectal cancer risk [20].

The *GSTM1 *and *GSTT1 *polymorphisms were unrelated to colorectal cancer risk singly or in combination in the present study. *GSTM1 *null genotype was associated with a small, statistically significant increase in the risk of colorectal cancer in some case-control studies [21,32], but not in several other studies [20,28-31]. Likewise, the previous findings on *GSTT1 *null genotype and colorectal cancer are inconsistent. A meta-analysis based on 11 studies reported a small increase in colorectal cancer risk associated with *GSTT1 *null genotype, but the results of these studies were highly heterogeneous [24]. Most of the previous studies found no increase in the risk of colorectal cancer in individuals with the combined null genotype of *GSTM1 *and *GSTT1 *[21,28,31]. On the other hand, a 5-fold increased risk of colorectal cancer was reported for simultaneous carriers of both *GSTM1 *and *GSTT1 *null genotypes in a study of 144 cases and 329 healthy controls in Spain [43]. In that study [43], *GSTM1 *and *GSTT1 *null genotypes were also statistically significantly associated with 1.9-fold and 3.6-fold increased risks, respectively. Frequencies of *GSTM1 *and *GSTT1 *null genotypes vary with different populations [22], but the difference in genotype distribution does not seem to explain the different results. The combined null genotype of *GSTM1 *and *GSTT1 *accounted for 24% among controls in the present study and for 7% in the Spanish study [43]. The statistical power was obviously greater in the present study than in the Spanish study.

At least six case-control studies have examined the relation between *NQO1 *Pro187Ser polymorphism and colorectal cancer [27], and only one study, which included 371 cases and 415 healthy controls in the Netherlands, showed a statistically significant increase in the risk associated with the variant 187Ser allele [44]. On the other hand, homozygotes of the *NQO1 *187Ser allele was associated with a 2-fold increase in the prevalence odds of colorectal adenomas in the United States [34]. In that study [34], individuals having both CYP1A1*2C and *NQO1 *187Ser variant alleles showed a significantly increased risk, particularly among heavy smokers. The present findings showed neither an increased risk of colorectal cancer in relation to the composite of *CYP1A1 *variant allele and *NQO1 *187Ser alleles nor an interaction between the composite genotypes and smoking.

A unique finding in the present study is that CYP1A1*2A or CYP1A1*2C allele was associated with a decreased risk only in individuals with *GSTT1 *non-null genotype. Interpretation of these findings is rather difficult, particularly because the association was confined to men. Available evidence suggests at least a secondary role of the *CYP1A1 *polymorphisms for increased risks of smoking-related cancers although the association between these polymorphisms and enzyme activity or property remains controversial [16]. Two case-controls studies of smaller sizes previously examined the combined effect of CYP1A1*2C and either *GSTM1 *[20,21] or *GSTT1 *[20] null genotype, showing no interaction between the two. The present findings on *CYP1A1 *and *GSTT1 *polymorphisms in combination may be due to chance, and need to be consolidated in further studies.

The use of community controls, the large number of subjects, and ethnic homogeneity of the study population were strengths of the present study. The statistical powers were fairly large except for CYP1A1*2C polymorphism. The powers of detecting an OR of 1.5 for variant homozygotes compared with wild homozygotes (two-sided α = 0.05) were 0.71 for CYP1A1*2A, 0.42 for CYP1A1*2C and 0.69 for *NQO1*, and the corresponding values for null versus non-null genotype were 0.96 for *GSTM1 *and 0.93 for *GSTT1*. There were several limitations to be discussed. The participation in the interview was not as high in the controls (60%) as in the cases (80%). We had no information as to the difference between participant and non-participant controls with respect to smoking history. The overall participation for genotyping was rather low (65% in cases and 56% in controls). Although older persons and women were less likely to give consent for the genotyping, there was no difference between those who gave consent and those who did not in terms of smoking, residence area, and alcohol use [45]. A retrospective assessment of cumulative exposure to cigarette smoking is subject to inaccuracy, and may have been biased because interviewers had known case-control status. Lifestyle factors were assessed for different time periods in the past for ease of recalling. This may have caused inaccuracy to different extents for the covariates, leaving different magnitudes of residual confounding. It is known that *GSTM1 *and *GSTT1 *genes contain nonsynonymous SNPs which may modify the enzyme activity [46], but these SNPs seem to be of little relevance in Asians as well as Caucasians [47]. Finally, although cases with familial adenomatous polyposis were not included, other hereditary colorectal cancers were not specifically ascertained in the present study. However, in the analysis excluding 16 cases and 40 controls aged <40 years, the results were essentially the same as those described above.

## Conclusions

The present study showed a moderately decreased risk of colorectal cancer, especially of colon cancer, in individuals with a light exposure to cigarette-smoking. A high exposure to cigarette smoking was associated with an increased risk of rectal cancer. None of the genetic polymorphisms relevant to the metabolism of tobacco carcinogens showed a measurable association with the risk of colorectal cancer. The observed interactions between *CYP1A1 *and *GSTT1 *polymorphisms warrant further investigation.

## Abbreviations

CI: confidence interval; CYP: cytochrome P-450; GST: gluthathione S-transferase; NQO1: NAD(P)H:quinone oxidoreductase 1; OR: odds ratio; PAH: polycyclic aromatic hydrocarbon; PCR-RFLP: polymerase chain reaction-restriction fragment length polymorphism.

## Competing interests

The authors declare that they have no competing interests.

## Authors' contributions

HN performed data analysis and prepared the draft. SK was in charge of the whole process including preparation of the manuscript. GY was in charge of DNA preparation and storage and analyzed data. KT and JN designed the study and conducted the survey. RM, MT, YK, YM, TO, KI, KF, TM, YY, KT, HI and RT contributed to study design and implementation of the survey. All authors read and approved the final manuscript.

## Pre-publication history

The pre-publication history for this paper can be accessed here:

http://www.biomedcentral.com/1471-2407/10/274/prepub

## Supplementary Material

Additional file 1 **Supplemental Table**. Effect modification of cigarette smoking on colorectal cancer risk associated with combinations of *CYP1A1 *and *GST *or *NQO1 *polymorphisms. Click here for file
